# Evolution of continents and the monsoon system: an interview with Gilles Ramstein

**DOI:** 10.1093/nsr/nwag294

**Published:** 2026-05-20

**Authors:** Yongyun Hu

## Abstract

*The monsoon system is driven by land–sea temperature contrasts. In the geological past, land–sea distributions experienced dramatic changes, with breaking up and reassembling. How do the land–sea distribution changes influence the monsoon system? This is an important question for understanding evolution, not only for the monsoon system but also for the Earth system. With this question, we interviewed Dr Gilles Ramstein, a worldwide leading expert in monsoon and paleoclimate. The interview starts from the modern monsoon to the paleomonsoon, and to the future monsoon. Dr. Ramstein also reviews his major contributions to the monsoon and paleoclimate fields. He also provides young scientists with great advice and warm wishes*.

## MODERN MONSOONS: REGIONAL VS. GLOBAL

***NSR*:** There may be thousands of papers on the topic of the monsoon system. Why are people so interested in monsoons?

***Ramstein*:** I think there are two major reasons. The first one, which is the most important one, is that billions of people live in the monsoon regions. Monsoons have such an important impact on our living environment. So, I think it is also kind of a demographic issue, and it is very important to be able to diagnose the evolution of monsoons. The second reason is for researchers. Monsoons are so fundamentally important. Monsoon is one of the climate systems with interactions between oceans and continents. A very fascinating question is to understand the evolution of the monsoon system at different timescales.

Paleomonsoon is a very attractive issue, and it becomes more and more attractive. It’s for sure that studying paleomonsoons benefits making better monsoon prediction for the future.

***NSR*:** There are six regional monsoons at present. What are the different characteristics between these regional monsoons?

***Ramstein*:** For the present day, there are two huge systems of monsoons: the African one and the Asian one. Other monsoon systems are also important, and they have big differences. Of course, there is the concept of the global monsoon. But in the history of the monsoon system, the concept of regional monsoons is quite different from the global monsoon. I will come back to the global monsoon concept later.

**Figure 1:**
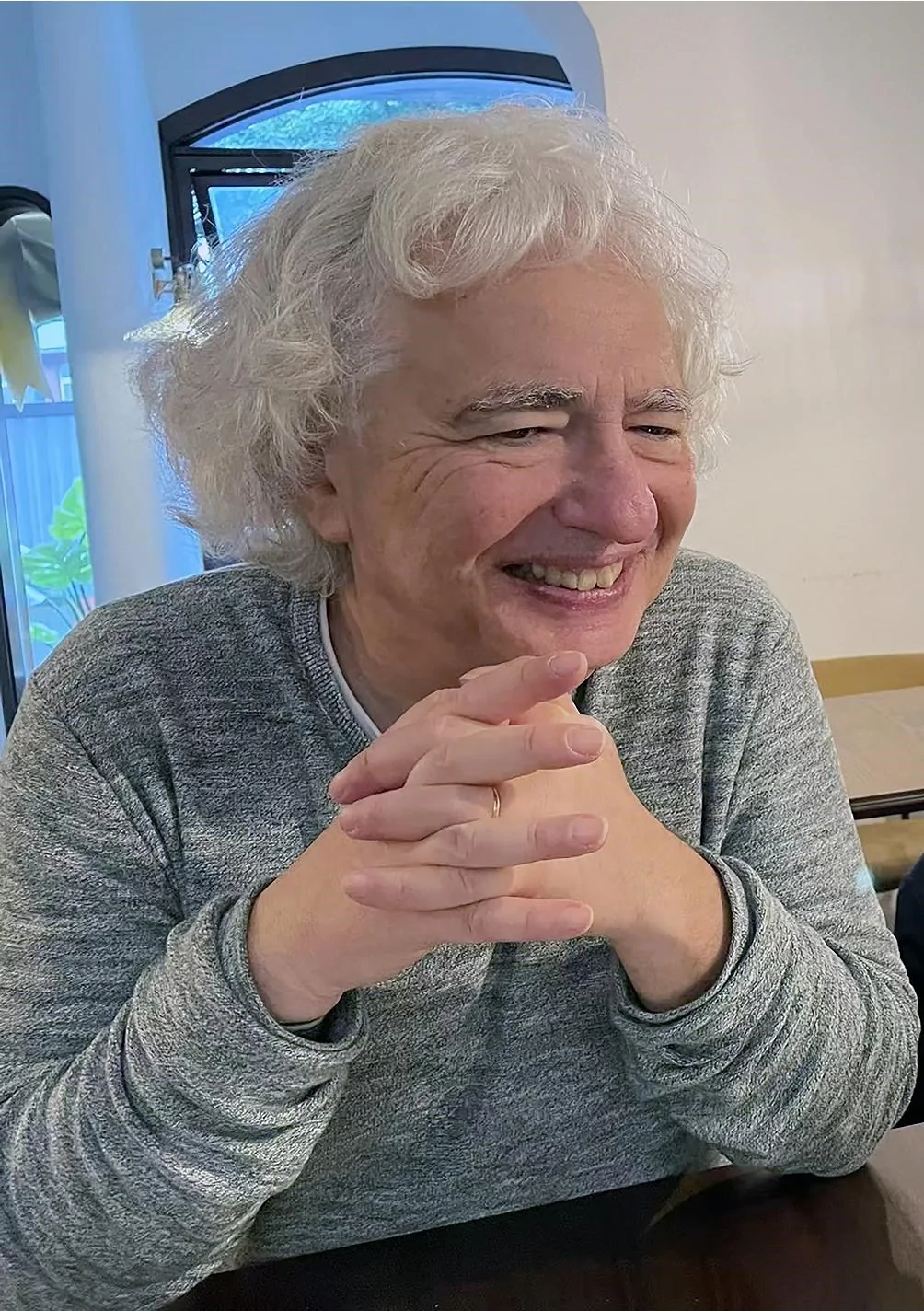
Prof. Gilles Ramstein. (*Courtesy of Ning Tan*)

A very fascinating question is to understand the evolution of the monsoon system at different time scales.

—Gilles Ramstein

The evolution of these regional monsoon systems is quite different. It is true not only for the past, but also for the future. This monsoon system is mostly driven by similar processes most of the time, but there are large differences between them. For instance, some of them are correlated with the El Niño–Southern Oscillation (ENSO). Some are related to freshwater from Greenland ice-sheet melting. If there is a large melting of Greenland ice sheets, it will have major impacts on the intertropical convergence zone (ITCZ), direct and large, so that it will push the ITCZ southward, and it will directly change the African South American monsoon, whereas this is not true for other regional monsoons.

***NSR*:** The concept of the global monsoon was proposed about two decades ago. What do you think about this concept? Is it better for describing monsoons?

***Ramstein*:** Yes, I think it is certainly an important concept, just not to be focused on only African and Asian monsoons. First of all, a good thing about the global monsoon concept for us is that we can use satellite data to study the monsoon system. As regional monsoons are unified with the global monsoon concept, we can see the interactions between different regional monsoon systems, and how they respond to the same processing, for instance, the increase of greenhouse gases. So, I think the concept is useful.

But for me, it is really important to understand specifically how each regional monsoon has evolved, and specifically how the evolution of different monsoon systems is driven by specific context, for instance the Asian monsoon is impacted by orogeneses of mountain ranges, whereas the African one is also modulated directly by freshwater from ice caps through glacial/interglacial episodes.

The regional monsoon is therefore dependent on timescales and paleogeographic evolutions. I will give only one example of this issue, which also pinpoints our capability to correctly capture monsoons with our models. At the end of the last century, in the 1990s, because of Kutzbach and Ruddiman’s tradition, we were upset by the onset of the Asian monsoon or the Indian monsoon. They claimed that the onset of the Asian monsoon was related to the uplift of the Tibetan Plateau. After that, a lot of progress was made. It is really due to our Chinese colleagues, so that we know much better than before the regional context of monsoon evolution. But does it mean only evolving the Tibetan Plateau uplift? It is not a simple question.

In the paleoclimate study, the most difficult parameter to infer is the value of elevation of the Tibetan Plateau. So, many Chinese modelers and people performing proxy data research try to get a better understanding of this. And this is something specific to each system, and it is not the same for the Australian monsoon or the South American monsoon.

But there are other problems. For the Asian monsoon, especially the East Asian monsoon, this is very important to me. I work on the onset of the East Asian monsoon, and Zhongshi Zhang worked on the African monsoon. Our work emphasized and quantified the different impacts of Tethys shrinkage and the Tibetan Plateau on the East Asian summer monsoon. But then after my work, maybe 15 years later, Zhongshi looked at the impact of the Tethys distribution. He found that the shrinkage of the Tethys played an equally important role in the African monsoon. Therefore, the shrinkage of the Tethys is important not only for the Asian monsoon but also for the African monsoon. This completely changed the hydrology.

So, my feeling is that when you understand a regional monsoon system specifically, you cannot avoid its global behavior. In this sense, it is the concept of the global monsoon.

## PALEOMONSOON

***NSR*:** The traditional view is that the monsoon system is driven by land–sea thermal contrast. How do you think this impacts the continental evolution of monsoons in the geological past?

***Ramstein*:** To make it understandable by people, it is very easy; for instance, I very often explain to people that about 40 million years ago, there was the huge Tethys over the present-day Eastern Europe and Western Asia. Upon this huge sea background, the summer would be cooler and the winter would be warmer, and so the monsoon system would not be so strong.

This is something you can understand because people can understand that when there is a large temperature gradient between continent and ocean, there will be a strong monsoon. And when there is not such a big temperature gradient, the monsoon system would not be so huge. Thus, it is really important to explain to people. However, it is a little bit simplistic. It is not only the temperature contrast between continent and ocean that can completely explain the driving forces. It is much more complex than this.

To explain it to people and to make it understandable, land–sea thermal contrast works. But in reality, the monsoon system is much more complex, for instance, the East Asian monsoon, and the Indian monsoon. It is very dependent on the topography because the spatial distribution of precipitation is drastically different, depending on the elevation. Both the elevation and the topography play important

…when you understand a regional monsoon system specifically, you cannot avoid its global behavior.

—Gilles Ramstein

For the African monsoon, it is not the North-South thermal contrast. It depends on a lot of atmospheric circulations. There are also some important feedbacks.

—Gilles Ramstein

roles in the monsoon system. It’s not only the term of thermal gradient that is good for scholar books, but for research, we really need to go deeper to have a better understanding and to capture more processes that can explain the spatial pattern and the duration of monsoons.

***NSR*:** Prof. Bill Boos at the University of California at Berkeley argued that land–sea thermal contrast is not accurate terminology in describing the driving force of monsoons. Instead, he suggested that moist static energy is the driving force. What do you think about this?

***Ramstein*:** I think it is absolutely clear. For instance, in France, to explain extreme events of rainfall, people are very fascinated by the fact that when we get extreme rainfall, which we call ‘Seven Events’, the Mediterranean area becomes extremely humid. The humidity of the air is very high because the surface of the Mediterranean Sea at the end of the summer is so hot, and you get a huge quantity of water vapor. This is the fundamental factor.

Water vapor is advected, and the first month with that humidity is July in France for this event, and you get a huge amount of rainfall. Can we explain it by the thermal gradient? The fact is that during the rainfall, humidity is the most important factor. I think you are right. The thermal contrast is a simplified view, as I said.

If you want to go a little bit deeper to explain the pattern of the precipitation, the duration, and its spatial and temporal structures, it is more complex than land–sea thermal contrast between ocean and continent.

***NSR*:** But some others still think land–sea thermal contrast is the driving force, whereas the moisture energy view is a kind of diagnosis.

***Ramstein*:** It really depends on what you look at. If you get a view as a mechanistic and energy guy, you will be interested in knowing where the energy is to disperse to, and of course, then you have to account for the budget of the latent and sensible energy, or the available energy, and you will get a different view.

I would think that both are true. It just depends on whether you are more interested in the energy contained in the system and its dispersal, or you are looking at me, as a fellow climate guy, and you understand the framework in which a monsoon occurs. If you are interested in the framework, when the monsoonal winds blow out, of course, it is quite dependent on the thermal contrast.

***NSR*:** To my understanding, the Asian monsoon is more related to land–sea thermal contrasts. What I mean is the North–South land–sea thermal contrast. But for the African monsoon, there is no such thermal gradient.

***Ramstein*:** There is a bit of thermal contrast between the North Atlantic Ocean and North Africa that can explain a lot of them. For instance, Zhongshi Zhang showed, through experiments from the late Eocene to the Paleocene, that the shrinkage of the Tethys completely changed the hydrological cycle and started the onset of the dry Sahara. Of course, there are linkages between the topography and the thermal contrast for the African monsoon. But it is much more complex. If it was so simple, all the models would predict the same thing for the 21st century, but they don’t. That means that it is not so simple, only by the land–sea contrast in summer.

For the African monsoon, it is not the North–South thermal contrast. It depends on a lot of atmospheric circulation. There are also some important feedbacks. For instance, my colleague Pascale Braconnot worked on the mid-Holocene monsoon; she proved that there is a lot of feedback to try to capture the real intensity of the African monsoon. One example of such feedback is by vegetation and lakes. These are certainly important.

In fact, the retreat of the Tethys is not only important for the East Asian monsoon you mentioned, but also for the African monsoon and the hydrology of North Africa.

—Gilles Ramstein

**Figure 2:**
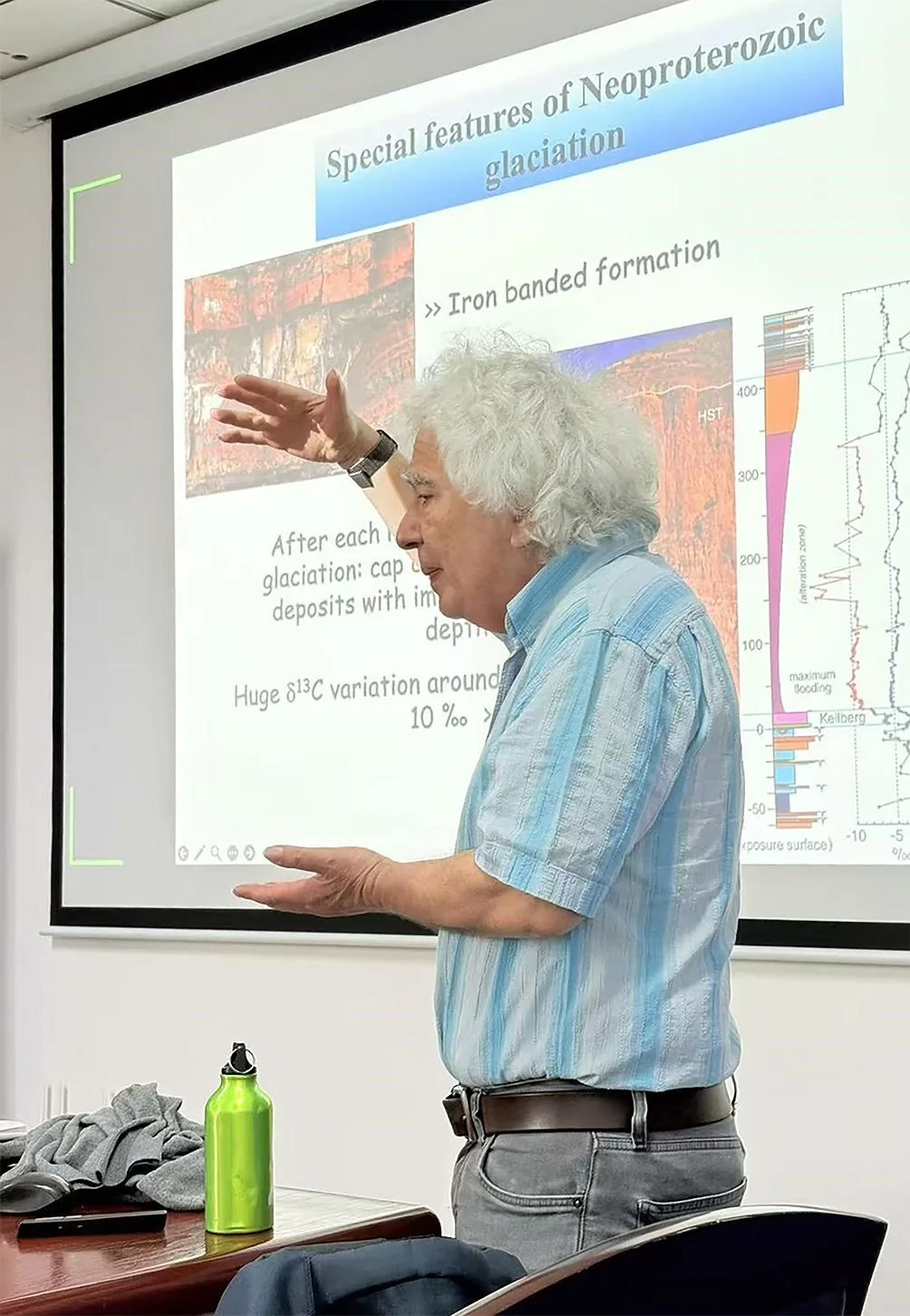
Prof. Gilles Ramstein giving a talk on the Neoproterozoic Snowball Earth at the Institute of Geology and Geophysics of the Chinese Academy of Sciences in 2025. (*Courtesy of Ning Tan*)

***NSR*:** From your point of view, what are the major areas of progress in monsoon research? Let us say from Kutzbach’s time or earlier time.

***Ramstein*:** Well, I know these guys, Ruddiman and Kutzbach, and they are good researchers. They have had really great ideas. I will not forget that when they began to work on monsoons, they only had the atmospheric-only model, and they got only very crude representations of the spatial resolution using atmospheric models at low resolution. Now, about 40 years later, it seems completely incredible.

Major progress has been made since then. Firstly, now, Earth system models (ESMs) are able to include not only the atmospheric component but also ocean and vegetation components and feedback. Secondly, using high-resolution models we are now able to better capture the topography, which is important for monsoon representation. It is progress that has been made by colleagues thanks to more sediment cores, which better document the evolution of the monsoon. Paleo-elevation reconstruction is so difficult. But now, we are much more able to understand the history of mountain ranges; therefore the models we use are no longer atmosphere-only models. They are coupled atmosphere–ocean ESMs, which include vegetation and even include δ^18^O. With these models, we can understand many things. So, from this point of view, it is extraordinary for me to see the progress we have made.

However, it’s not the end of the research because if we were so good, we should be able to diagnose what the response of the monsoon system is to just the doubling of CO_2_. It is far from easy, because first, there is not only doubling of CO_2_, but also changes of land use and increase of aerosols.

On the one hand, you get incredible progress on understanding the monsoon system and its interaction with the ocean and the atmosphere. On the other hand, there is still a lot of progress to be made to be able to capture its good reliability, because these results are so important for billions of people for the future.

***NSR*:** I think one of the major areas of progress in monsoon research, especially for the East Asian monsoon, is the work

by Zhongshi Zhang and you. In that work, you argued that the retreat of the Tethys is equally important as the uplift of the Tibetan Plateau to enhance the East Asian monsoon.

***Ramstein*:** I think it was a very big point at that time. In fact, the retreat of the Tethys is not only important for the East Asian monsoon you mentioned, but also for the African monsoon and the hydrology of North Africa. For the East Asian monsoon, it is indeed one of the major components, just like the uplift of the Tibetan Plateau.

***NSR*:** You have mentioned that paleogeography is important for paleomonsoons, such as the shrinkage of the Tethys. What about the impact of CO_2_ or climate change on paleomonsoons?

***Ramstein*:** How will the monsoon system react to CO_2_? If you look back at the past, is CO_2_ a very important key factor for paleomonsoons? You will find the publications by Alex Farnsworth, and your work. These showed that, in fact, it is mostly the changes in paleogeography that drove the monsoon system over tectonic timescales, rather than CO_2_. I believe that it’s true; I think that paleogeography is the key factor in driving paleomonsoon evolution at the geological scale. CO_2_ is important, but the first player is certainly the continental distribution on geological timescales. This is why I think for geological timescales there are two major geological events, the shrinkage of the Tethys and the uplift of the Tibetan Plateau, which are crucially important for Asian monsoons. It is much more complex than the mountain range. Moreover, thanks to the work of Ran Zhang, Yan Zhao and other colleagues, their work demonstrates that other mountain ranges are also playing a role in Asian monsoon evolution. We know now the two geological factors that changed the most. This is really very important. CO_2_ is also important. In contrast, CO_2_ evolution for the present day is so fast that it will imprint deep climate change and modify the monsoon system.

But I do not believe that it is so important for geological timescales. Coming back to the geological timescale, seaway changes certainly also had impacts on monsoons.

***NSR*:** We also have two recent papers that demonstrated that over tectonic timescales continental evolution governs the global monsoon system, and we also showed that the monsoon system demonstrated super-cycles, which was driven by the super-continental cycle.

…it is mostly the changes in paleogeography that drove the monsoon system over tectonic timescales, rather than CO_2_.

—Gilles Ramstein

If there is a decrease of the AMOC, it will impact global climate and will push the intertropical convergence ITCZ southward and consequently impact on monsoons and areas where billions of people are living.

—Gilles Ramstein

***NSR*:** What are your thoughts on the linkage between monsoons and human evolution and dispersal? You already mentioned some of it.

***Ramstein*:** In fact, I have worked a lot on this during the past few years. I have got a big program to try to understand the records. The records show that at the beginning of the Neogene, about 23 million years ago (Ma), the apes lived in tropical forests. We have records of bones and pollen. We also know that at 17 Ma during the Middle Miocene Climate Optimum, they were able to get out of Africa by different ways and come to Europe. There are bones and pollen to show it. This was the first time for the apes to get out of Africa. It is really not random because the climate enabled tropical forests during the middle Miocene in South Europe. It was a good environment for them to live in, and some of these apes decided to go to Europe.

We also know that at 14 or 13 Ma when the cooling began again, there were no more tropical forests in Europe. You get no more pollen, no more bones of apes. There were two choices for apes: coming back into Africa or staying in Southeast Asia. We know that they migrated because the climate was cooling, and cooling again. Their niche was reduced and they reached Indonesia at the end of the Miocene, where they still live.

This dispersal is in fact completely driven by climate. It is not like *Homo sapiens*, who could adapt. The apes could not adapt to the cooling, and they had to live in tropical forests. With our climate simulation coupled with the vegetation model, we can simulate this dispersal. With my colleagues, we have worked hard on this to understand this dispersal.

## FUTURE MONSOONS

***Ramstein*:** I believe that for future climate, CO_2_ is still very important. I will just give two examples, which come from the Chinese publications. The impact of land use is an important factor. The impact of our human activities results in an emergent forcing factor. This is a new factor and new force for which we have no analogy from the past. We are still working on these factors to gain better knowledge.

***NSR*:** From your point of view, what are the important directions of monsoon research we should work on?

***Ramstein*:** I also work on another topic: from now through the next few decades or a century, we can expect that ice sheets will be partially melting, but not linearly. If you look at the record of glaciation, you can see that the deglaciation from 19 to 9 ka caused a sea level rise of 120 m. We have very good records of sea level. We perfectly know that it is not a linear process. The sea level rising during our period is accelerated. I think the most accelerated rate was 3.7 mm for years to decades. But today, it is already 4.2 mm. There is the maximum acceleration of sea level rise we have ever seen during the Holocene. So, I think that it is important to account for that fact if you get accelerated melting of Greenland and Antarctic ice caps.

Especially for Greenland and West Antarctica, what will happen is a serious decrease of the Atlantic Meridional Oceanic Circulation (AMOC). This is what we are sure of because paleoclimate data show that there were many events of large surges of icebergs. If there is a decrease of the AMOC, it will impact global climate and will push the ITCZ southward and consequently impact on monsoons and areas where billions of people are living. This means that for future research, I think we have to take care of the melting of the ice sheets.

## MAJOR ACADEMIC CONTRIBUTIONS

***NSR*:** You are a world-renowned paleoclimatologist. How do you summarize your scientific career? What are the most important accomplishments of your career?

***Ramstein*:** I don’t know if I am a world-renowned researcher. But I can tell you what, for me, the major achievements are in three areas in which I have produced something new. The first one is the Snowball Earth. This is very important for me. The major discovery and the explanation we were trying to understand are: Why can a Snowball Earth occur? How is it possible? Why did it last millions of years? Why did it last so long on Earth?

On this topic, I think, thanks go to my students; I have to say that, and this is very important. I always get very good students, and thanks to them, I can make some progress.

For the Snowball Earth, we tried to understand why a snowball occurred during the Neoproterozoic. There was work with Yannick Donnadieu and all his colleagues. I think we solved this problem. And then we also understood, why the end of the Snowball Earth is looking like glaciation and interglaciation. So, on this topic, I think I have a little bit of a contribution.

Another one is associated with collaboration with geologists and sedimentologists. I visited the Institut de physique du globe de Paris (IPGP) in Paris, which is similar to the Institute of Geology and Geophysics, Chinese Academy of Sciences (IGGCAS) in Beijing. There was a researcher called

So, my opinion is that it is very important to keep fundamental research going. If there is no fundamental research, the climate impact research will be really bad, because the fundamental research is a reservoir of ideas.

—Gilles Ramstein

Jean Besse, and he and his geologist colleagues mapped the shrinkage of the Tethys from the Cretaceous to the Miocene. They published, for the first time, a documented atlas. They talked to me: it is quite strange, we worked a lot on this topic, but no modelers take care of it. Then, I said, it is very simple, and I can do it if you want.

It is not because of me, but it is truly because I listened to these data people. With their paleogeographic maps, and with my colleague Frédéric Fluteau, I can simulate the climate during the Cenozoic, and we can quantify some scenarios, especially the impacts of the shrinkage of the Tethys on the climate and monsoons. I also work on different aspects of climate evolution during the Cenozoic related to the closing and opening of seaways as a major forcing factor of ocean dynamics, the impact of climate and hydrology on pre-human dispersal, etc.

I also work on future climate in terms of thresholds. For instance, I work a lot on the Henrich events, and what happens when you get a large surge of icebergs: what will happen to the climate? And how will oceanic and atmospheric dynamics respond to it? How will sea level rise? I have worked hard on these topics.

As I told you, I have also worked a little bit on a completely different topic, which is the dispersal of vector-borne disease for the 21st century with classical Intergovernmental Panel on Climate Change (IPCC) scenarios. I also work with scenarios that account for partial melting of the Greenland ice sheet and show its impact on the AMOC and deep modification of hydrology in the monsoon area, which led to dispersal of malaria. This is also quite important for me. When we published this paper with my colleague Cyril Caminade and Alizée Chemison on what will happen with the accelerated melting of Greenland, we showed that there will be migration, not only upon the coastal area, but also on the monsoon region.

Cyril Caminade is an expert on vector-borne diseases and told me that it is not only migration of humans, but also migration of vector-borne diseases. I asked him what it is: ‘Please explain malaria to me’. This researcher said that, according to the IPCC scenarios, the next century will become so hot. In low-land Africa, even mosquitoes cannot stay there, they would die, so they will also migrate to the Eastern Plateau of Africa, where they can get better precipitation and temperature. With the IPCC scenarios, malaria will move to the Eastern Plateau of Africa.

I think this is quite important because now, in France, we get many vector-borne diseases. This is due to climate change because it provides appropriate conditions for some vector-borne diseases to migrate and develop. They changed the area, and there are new potential areas for them.

I published a paper on this, and Cyril also published a paper on this topic; we worked together to see if there is agreement. It demonstrated that climate change will also change malaria’s distribution and push malaria to Eastern Africa, whereas our simulation accounting for partial melting of Greenland ice sheet has consequences to produce migration toward South Africa.

I think this is also a little contribution to future climate change. I am very glad to be able to thank my paleoclimate study that provides some scenarios for preventing diseases. Such studies could be very useful to prevent, or adapt for, the dispersal of vector-borne diseases due to rapid climate changes.

## ADVICE AND WISHES TO YOUNG SCIENTISTS

***NSR*:** What would you like to say to young people to inspire their interests in the paleoclimate?

***Ramstein*:** I don’t know how to say that to young people in China, because the academic system is different from the French one. In France now, when good students want to do some research, they come to me and say: ‘Gilles, I want to work on Snowball Earth’. Maybe I would tell them: ‘Research on the Snowball Earth is very interesting, but that research can wait for a little bit. You may want to work on something to be useful, such as the impacts of climate change, and my knowledge on oceanic and atmospheric dynamics can help.’

But there is one thing that we have to keep in mind, and it is very important. If everybody wants to work on climate impacts, we also need to work on fundamental research, especially the key questions of fundamental research. Here I only give two examples. First, there is the possibility of destabilization of ice sheets, which impacts on not only Greenland and West Antarctica ice sheets, but also global climate, due to the surge of icebergs that would kill the North Atlantic Ocean. This is a kind of paleoclimate research, and it is really fundamental research.

Another example is linked to the ocean. Is global mean sea level just the consequence of global warming? Can it be an important forcing factor of climate change by itself? You can only test that for a large-scale circulation model. We noticed that the sea level rise was about 2 to 6 m for last interglacial period. If you account for this, it is a simulation work of the last interglacial period first performed and published by Zhang *et al.* using the NorESM1-F model. To get better simulation results, one can only come to fundamental research. So, my opinion is that it is very important to keep fundamental research going. If there is no fundamental research, the climate impact research will be really bad, because the fundamental research is a reservoir of ideas.

I get the feeling that in China a good advantage is that you have a long-term thought, and you know where you will go.

—Gilles Ramstein

***NSR*:** Today, young scientists have pressures from both publication and research funding. Any advice for them?

***Ramstein*:** Yes, to do paleoclimate research, you need some money, a little bit of money to go here and there, to provide new sediment records, and to perform modeling. This is indeed a real problem to get money from funding agencies, at least in my country. For young scientists, it is a huge pressure for them both to be able to get successful projects to be funded and to have publications to finally get a permanent position. I was very lucky because 40 years ago, at least in France, there was the pressure of publication, but it was not so huge. It was not so difficult to get projects supported too.

But now, even me, as a senior scientist, and you think I am a worldwide-known researcher, I have to fight to get the money that allows me to do research for successful projects. I have lost a lot of time on it. I am good at doing research, but I am not very good at finding money. So, I think it is not so good for old people like me. Nevertheless, for young people who are just beginning their research work, the pressure to secure funding is even worse. It is better to create more opportunities for them.

In fact, I have the feeling that in France one of the problems is that there is no real long-term plan. People are fighting for what will happen in politics in the next two years. I have come to China many times. I get the feeling that in China a good advantage is that you have long-term thinking, and you know where you will go. So, I think there is actually a better situation in China than in France for funding. On the other hand, although there is more money in the system in China, there is really a serious pressure for publication on young scientists who work on fundamental research, because you have so many researchers.

Publication is important. However, this idea to publish many papers is not reasonable in Europe, and it is not so good for research, because research needs time. You can think about a hypothesis for many years and get good and reliable results. If you publish a paper every 6 months, the results could be proved wrong 6 months later. It is nonsense for me, because people are too stressed out and finally publish not-so-appropriate papers. It is good to keep very good scientists in the system. It is very important to mostly secure fundamental research and to attract excellent students to provide good conditions in terms of salaries and funding for long-term projects. We need to think about this issue seriously.

